# Young onset dementia: Public involvement in co-designing community-based support

**DOI:** 10.1177/1471301218793463

**Published:** 2018-08-19

**Authors:** Andrea M Mayrhofer, Elspeth Mathie, Jane McKeown, Claire Goodman, Lisa Irvine, Natalie Hall, Michael Walker

**Affiliations:** Centre for Research in Public Health and Community Care, University of Hertfordshire, Hatfield, UK; School of Nursing and Midwifery, University of Sheffield, Sheffield, UK; Centre for Research in Public Health and Community Care, University of Hertfordshire, Hatfield, UK; Norwich Medical School, University of East Anglia, Norwich, UK; Centre for Research in Public Health and Community Care, University of Hertfordshire, Hatfield, UK; Hertfordshire Partnership University Foundation Trust, Logandene, Hertfordshire, UK

**Keywords:** Age-appropriate dementia care, continuity, co-production, young onset dementia

## Abstract

Whilst the support requirements of people diagnosed with young onset dementia are well-documented, less is known about what needs to be in place to provide age-appropriate care. To understand priorities for service planning and commissioning and to inform the design of a future study of community-based service delivery models, we held two rounds of discussions with four groups of people affected by young onset dementia (n = 31) and interviewed memory services (n = 3) and non-profit service providers (n = 7) in two sites in England. Discussions confirmed published evidence on support requirements, but also reframed priorities for support and suggested new approaches to dementia care at the community level. This paper argues that involving people with young onset dementia in the assessment of research findings in terms of what is important to them, and inviting suggestions for solutions, provides a way for co-designing services that address the challenges of accessing support for people affected by young onset dementia.

## Introduction

Current estimates of people living with young onset dementia (YOD) (diagnosed at age <65 years) in the UK are around 42,500 (Prince et al., 2014). On average, time from early symptoms to diagnosis is 4.5 years (Draper et al., 2016; Van Vliet et al., 2013). Diagnosis is often delayed for a variety of reasons that include the heterogeneity of symptoms (Picard, Pasquier, Martinaud, Hannequin, & Godefroy, 2011), an overlap of symptoms that are not necessarily dementia specific (Suárez-González, Henley, Walton, & Crutch, 2015), atypical dementias (Kilarski et al., 2015), differing standards in diagnosing dementia (Rosness, Engedal, & Chemali, 2014) and individuals’ attitudes to screening (Bunn et al., 2012; Martin et al., 2015). There is a long and cumulative literature on the difficulties and challenges of living with YOD. For example, younger individuals are likely to still be in employment (Alzheimer’s Society, 2015; Chaplin & Davidson, 2016; Picard et al., 2011; Ritchie, Banks, Danson, Tolson, & Borrowman, 2015; Robertson, Evans, & Horsnell, 2013) and carry financial responsibilities for their families. They have to give up work (Rayment & Kuruvilla, 2015; Roach & Drummond, 2014; Tyson, 2007), often due to reduced job performance (Richardson et al., 2016). Spouses may have to reduce their working hours to take on a caregiving role (Shnall, 2015) or, alternatively, may have to find employment to support the family financially (Kinney, Kart, & Reddecliff, 2011). Families struggle to find advice and support in relation to pension payments, benefits, insurances and longer term arrangements (Gibson, Anderson, & Acocks, 2014; Roach & Drummond, 2014; Shnall, 2015; Wheeler et al., 2015), especially if they have teenage children or look after an ageing parent (Gibson et al., 2014). They also tend to enjoy higher levels of physical fitness than older people (Roach, Drummond, & Keady, 2016; Tolhurst, Bhattacharyya, & Kingston, 2014) and seek to continue and actively pursue their interests and hobbies (Armari, Jarmolowicz, & Panegyres, 2013; Brown et al., 2012; Cabote, Bramble, & McCann, 2015; Dementia Pathfinders, 2016; Ducharme, Kergoat, Antoine, Pasquier, & Coulombe, 2014; Ducharme, Kergoat, Coulombe, et al., 2014; Ducharme et al., 2016; Gibson et al., 2014).

Services designed for older people such as day care centres for older people with dementia therefore do not ‘fit’ younger people. Being exposed to people in advanced stages of dementia can be quite disturbing for those who are at the beginning of their journey and in the process of adjusting to their diagnosis whilst still in a very active phase of life (Millenaar et al., 2016). As expressed recently by a young person diagnosed with dementia: “We are told to give up our pre-dementia diagnosis lives and to get acquainted with age-care services” (Swaffer, 2018). Age therefore is a factor when designing support for younger people diagnosed with dementia. Their support needs are different to the care required by older people in their 80s or 90s when functional decline as a feature of normal ageing is worsened by dementia.

This study aimed to establish what was known about the range of post-diagnostic interventions designed for people diagnosed with YOD and their family caregivers, which elements of support were perceived as most effective by people affected by YOD, and how age-appropriate services needed to differ from generic dementia services offered to much older populations. Some findings were reported previously (Mayrhofer, Mathie, McKeown, Bunn, & Goodman, 2017). This paper focuses on suggestions made by people diagnosed with YOD and their caregivers in the two rounds of discussions held around different approaches to service design.

## Methods

**Figure 1.**
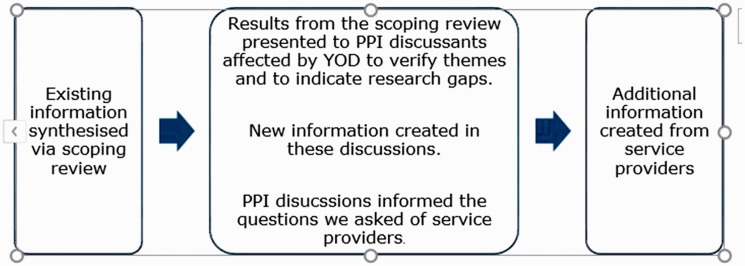
The role of PPI discussion groups.

We undertook a scoping review of the literature on what was known about the range of post-diagnostic services and discussed these findings with four groups of people affected by YOD. These discussion groups were not research study participants, but patient and public involvement (PPI) contributors and are referred to as PPI discussants (INVOLVE, 2012). The purpose of these discussions was to establish whether the themes presented in national and international literature reflected their experience of service provision, to highlight research gaps and to inform questionnaires for service providers. This approach is useful for commissioners and practitioners when assessing the relevance and applicability of evidence that may come from diverse sources and settings and is synthesised over extended periods of time (Bunn et al., 2015). Researchers met PPI discussants via existing Alzheimer’s Society support groups and had a number of reflective conversations about the findings of a scoping review around post-diagnostic services for younger people with dementia (Figure 1).

### Scoping of the literature

Our approach was based on the methodological framework for scoping reviews (Arksey & O’Malley, 2005) in relation to identifying and selecting relevant studies, charting the data, optional consultation, and collating and summarizing the results.

#### Identifying and selecting relevant studies: Data collection and scoping process

To establish what was known about the range of post-diagnostic services, and which components of care were perceived as age-appropriate and meaningful, we undertook a scoping review on service provision for younger people with dementia. We searched PubMed, Cinhal, Scopus, Ebsco Host and Social Care Online, used Google Scholar, undertook lateral searching, hand-searched dementia specific peer-reviewed journals and carried out online searches for grey literature published by the National Health Service and third-sector organisations (March–June 2016). The main inclusion criterion was for the literature to be YOD specific. Papers and reports that focused on dementia more generally were excluded. Search terms used are shown in Box 1.

Box 1 Search terms.

**Table:** 

“Young onset”, “early onset”, dementia, Alzheimer*, YOD, assessment, diagnosis, “post-diagnosis”, “post-diagnostic”, service*, “service provision”, support, intervention*, “care need*, “dementia care”, NHS, hospital, charity, “third sector”, community (and various combinations of terms)

#### Involvement of people affected by YOD

To advise on how evidence about living with YOD was interpreted in the literature and to understand which of the services were perceived as most supportive and effective, we held two rounds of discussions with four groups of people affected by YOD in two disparate study sites in England. Access to existing YOD groups who meet once a month was facilitated by the Alzheimer’s Society. Three groups were comprised of a person living with dementia and their caregiver and were consulted as dyads. In one group, researchers had only access to family caregivers. The YOD groups were consulted as PPI contributors/discussants, also referred to as ‘experts by experience’, to shape the research (INVOLVE, 2012). PPI discussants and/or their family caregivers received information sheets prior to the meeting and, as a requirement of the Alzheimer’s Society, signed consent forms. We used a topic guide that stated the purpose of the meeting and the main question for discussion and ensured that there was enough time for questions to be asked (Brooks, Gridley, & Savitch, 2017; Giebel et al., 2017; Rivett & Rivett, 2017; Smith, Rossor, & Kotting, 2017). We did not ask for personal information, except for demographics such as gender, age, age at diagnosis and sub-type of dementia. Discussions were iterative. The first round of discussions reflected on interim findings of the scoping review. The second round discussed their views on what dementia care and support in their community might look like and what their priorities were. Two researchers took notes during the discussions. Information was grouped into themes and synthesised across the four PPI groups. The points raised by the groups echoed the topics of the scoping review and helped the researchers shape the systematic review. We returned a third time to meet with PPI discussants at the end of the study to provide feedback (Mathie et al., 2018). We discussed with the groups how their involvement had shaped the research and what the way forward might be in supporting younger people with dementia and their family caregivers. A written lay summary of study findings was provided to those attending the group. Additional copies were left with the groups for them to disseminate to people they thought might be interested in the study. We also posted printed copies to group co-ordinators for further dissemination.

#### Interviews with service providers

To understand how service provision for people with YOD is conceptualised by organisations, we undertook face-to-face and/or telephone interviews with members of staff of memory services and third-sector organisations that provided services in two disparate sites. Third-sector organisations were purposively sampled local and national charities that had been mentioned in the two rounds of discussions with those affected by YOD. Interview data were transcribed verbatim and analysed thematically (Mason, 2017) by three researchers (AM, EM and JMc) using qualitative data management software QSR NVivo version 11 (NVivo, 2015). Interview data from memory services are denoted by the key ‘MS’. The key ‘TSO’ refers to information and interview data from third-sector organisations.

#### General public and patient involvement

In addition to the discussion groups who commented on findings and services, we had Public and Public Involvement (PPI) input prior to commencing the study to ensure that the overall approach was clear and relevant. An Alzheimer’s Society representative diagnosed with YOD commented on the funding application. Two members of the University’s Patient & Public Involvement in Research Group, both of whom have personal experience of family members with dementia and are regular volunteers with the Alzheimer’s Society, joined the study’s advisory group for the duration of the project. They commented on the design of the questionnaire for service providers and on the lay summary of findings prior to dissemination. Minor adjustments were made to the lay summary.

### Ethics approval

The research protocol was reviewed and approved by the University of Hertfordshire Ethics (Protocol Number HSK/SF/UH/02540). The Alzheimer’s Society research support office facilitated access to people affected by YOD for our discussions.

### Findings: Summary of scoping review

The scoping review identified literature reviews of the experience of living with YOD (n = 12), systematic reviews (n = 5), peer-reviewed papers (n = 22), evaluations (n = 3), a guest editorial (n = 1) and reports published by charities and non-profit organisations (n = 6). The literature reviews and systematic reviews included more than 700 documents. Recurring, well-known and frequently reported themes related to the length of time it took to receive a diagnosis, to learn which support was available in the community locally and the need for specific support needs (Austin, O’Neill, & Skevington, 2016; Baptista et al., 2016; Ducharme, Kergoat, Coulombe, et al., 2014). Support needs were determined by the relatively young age at which individuals were diagnosed (Armari et al., 2013; Dementia Pathfinders, 2016), by pre-mature and unplanned retirement (Chaplin & Davidson, 2016) and by different and often rarer sub-types of dementia (Chemali, Withall, & Daffner, 2010). A cumulative body of literature emphasises the differences between age-appropriate support and dementia services for older people (Mayrhofer et al., 2017). Services were perceived as useful if they provided information about specific aspects of YOD (Carter, Oyebode, & Koopmans, 2018), signposted to different services in the community, addressed the person diagnosed with YOD and their caregiver as a dyad and were responsive to a person’s/family’s changing needs as the illness progressed (Westera et al., 2014).

Community-based support services that were felt to be effective further along the illness trajectory featured the following elements: they provided continuity, offered stimulation, preserved identity, dignity, personhood and agency (Carone, Tischler, & Dening, 2016; Ducharme et al., 2016), facilitated social connectedness and prevented families from becoming socially isolated (Roach & Drummond, 2014). Barriers to accessing such initiatives were a lack of knowledge that they existed, lack of transport or if caregiver respite meant that the younger person with dementia would have to spend time in a residential care home for older people (Gitlin, Marx, Stanley, & Hodgson, 2015). The scoping review showed that some high-quality support was provided, but its sustainability was determined by the way services were commissioned and funded. These were the findings we took to the PPI discussion groups.

## PPI contributions by people affected by YOD

Two rounds of discussions with four PPI groups consisting of people diagnosed with YOD and their caregivers were held in two sites in England in May and October 2016. Two researchers attended each of the groups (AM and EM; AM and JMc). Members of Alzheimer’s Society staff and volunteers were also in attendance. Of 31 discussants, 11 had a diagnosis of YOD and 20 were spousal caregivers (Table 1). Seven of 11 people with YOD were males and four were females. Types of diagnoses included Alzheimer’s disease, vascular/mixed dementia, Lewy bodies, semantic dementia, posterior cortical atrophy, Pick’s disease, and cerebral autosomal dominant arteriopathy with subcortical infarcts and leuko-encephalopathy (CADASIL). Three PPI discussants did not state the type of dementia. Two group members were older than 65 years when they received a formal diagnosis, but reportedly had been symptomatic for a considerable length of time prior to being diagnosed. The age range was from 48 to 70 years.

**Table 1.** PPI discussants.

	Group 1	Group 2	Group 3	Group 4	
Person diagnosed with YOD	0	6	0	5	11
Caregivers	8	5	2	5	20
Total					31

YOD: young onset dementia.

In the first round of discussions, a researcher presented a brief overview of the main themes that emerged from the scoping review, asked whether this matched people’s experience and invited comments as to people’s perceptions of service use. Discussants agreed with the main recurring themes presented in the literature but were keen to emphasise the significance of some, but not all of the findings. They stated that many community-based services were provided on a short-term basis only and therefore lacked continuity of care. Some services had long waiting lists and frequent staff changes. PPI groups brought new insights to research findings about the lack of continuity of services and stressed the challenge of having to re-orientate themselves, having to find new services and, in effect, having to return to their immediate post-diagnosis status. For people living with a diagnosis, this lack of continuity was not just a loss of a service but also a loss of contact with people whom they had just met, who were like themselves and could empathise. Sustainability of services, continuity of care and local, community-based, easily accessible social networks were particularly important for these groups who found mainstream dementia services inappropriate. The lack of advice and support around finances in unplanned, pre-mature retirement was discussed in the scoping review, but the PPI representatives identified this as a pressing service need and specific research gap that needed to be prioritised.

In order to shape the interview questionnaire for service providers, the second round of discussions revisited these issues and asked specific questions around which kind of community-based support could improve or modify services. One PPI discussion group stated that they had been giving this considerable thought as the earlier discussion had provided a platform and stimulus for thinking about what might be effective. They proposed a service model where a local branch of a charity (such as the Alzheimer’s Society) could act as an ‘anchor’ and become the go-to-place for advice around which other services and groups could self-organise. This was seen as likely to facilitate contact with other families affected by YOD who are scattered in their community. The local charity could be the hub for building their own social networks around shared interests, similar conditions and/or geographical proximity, all of which was represented as likely to support continuity of contact and peer support. One group mentioned the idea of ‘skills swapping’, for example, driving, offering transport to various events, or offering gender-specific support such as a woman accompanying another woman to go swimming or shopping for clothes, or a man taking another man to watch a game of football. Such activities presume knowing who else in their community is affected by YOD.

The groups offered a different perspective on what services should achieve. The aim was to become less dependent on ‘services’ as families learn to support each other, socialise with each other and help each other to live as well as possible. In these discussions, the service or centre could be a catalyst for self-organisation as well as a safety net for those who struggled to make connections. In the last PPI discussion group meeting we drew a diagram to capture discussants’ thoughts and comments.

**Figure 2.**
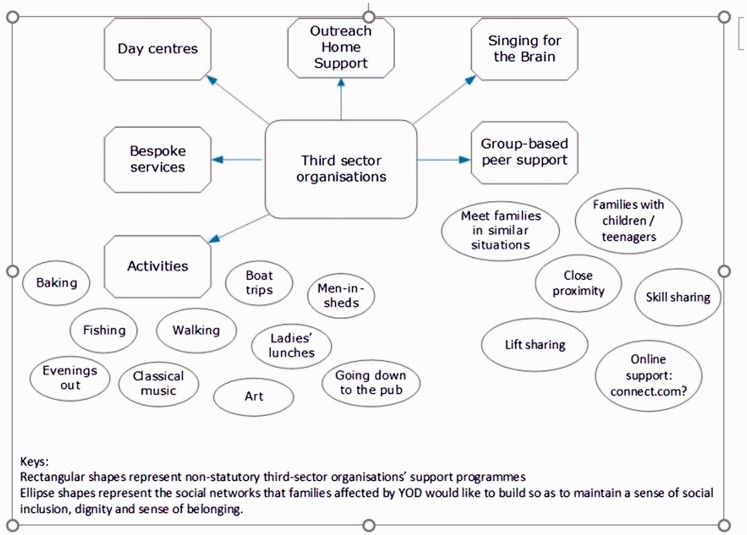
Developing support networks at the community level.

The rectangular shapes in Figure 2 depict a range of services offered by non-statutory third-sector organisations. These include day services for people with YOD, bespoke services, home support, various activities and group-based peer support. However, as pointed out in PPI discussions, a range of activities that people diagnosed with YOD would like to take part in are currently unconnected (depicted in ellipse shapes).

A recurring idea in the PPI discussions was the importance of enabling families in similar situations to support each other and potentially reduce the demand for overstretched community-based services. The value of family members being able to meet peers who faced similar issues and more generally to foster a sense of social inclusion and social connectedness was discussed as important, and a lack of knowledge of other families affected by YOD in their local area was seen as a significant barrier to building networks of informal support. It was suggested that organisations’ concerns about data protection could have unintended consequences of maintaining isolation. Discussants thought that one way of overcoming this could be for organisations to offer an ‘introduction service’, which people could opt in to. They also suggested the development of online resources designed to contact other families affected by YOD in the community or to find events. They were sophisticated in their understanding of what would and would not work. Whilst the scoping review had recognised the pressures on families, studies had not explored how agencies could mediate peer-to-peer support with different members of the family.

In contrast to the needs identified in the literature, discussants talked about organically developed community-based networks that are anchored by a core. They were pragmatic and recognised that the small numbers of people living with YOD in any area tended to work against the sustained funding of service-led provision. PPI discussants saw third-sector organisations as a focal point for advice and support, but equally importantly as advocates on their behalf to liaise with local clubs, restaurants and businesses to make their services to the public more accessible and dementia friendly. Concrete and practical suggestions included reduced rates for leisure centres for people with YOD who, though physically fit and active, found themselves retired pre-maturely. They suggested organising a ‘slow swimming lane’ during quiet times of the day, providing a closed-off room in a restaurant a few evenings per month and negotiating reduced rates for buses for families affected by YOD. This was seen as achievable because of the small and relatively inexpensive adjustments required for the small numbers of people with YOD they would apply to. PPI groups highlighted that literature had not investigated cross-cutting links between services or how this might foster group-based peer support.

PPI contributors were grounded in the realities of provision, and group discussions enabled judgements on the relative significance of research findings. Issues such as project-based commissioning, sustainability of services, continuity of care and waiting lists for community-based support were recognised and addressed. They were also recognised and addressed by service providers.

## Interviews with service providers

To determine service providers’ views on the themes identified in the literature and by PPI contributors we undertook face-to-face and/or telephone interviews with memory services (n = 3) and non-statutory service providers (n = 7) in two study sites in England. Two of the three memory services worked with a multidisciplinary team that included an Alzheimer’s Society support worker. Teams either helped the person diagnosed with YOD and their family carer directly or referred them to community-based services for support (MS01 and MS06). The third memory clinic had Alzheimer’s Society support staff as the ‘go to’ person and link between public sector and third-sector services and the community (MS08). However, this post was project-funded and discontinued after one year.

Interviews suggested that person-centred long-term relationships were more likely to be established if support workers remained closely involved with patients and their families in the longer term and did not withdraw services once a person reached the age of 65 years. As a clinician from a memory service observed:

A problem from a service provider’s (memory clinic) point of view is what to do when people get older. A service designed for younger people with dementia must either discharge people when they get to a certain age or end up being a service where people are on average not young. This is compounded by the age structure of dementia – exponentially increasing with age, so most people under 65 with dementia are only just under 65, that is, 60 to 64. The same goes for any specific age cut-off down to the age of 40 (according to Prince et al., 2014 figures). The very young are very rare, and the majority are just below the cut-off age for traditional ‘Old Age’ services. So if you cut off at 65 and actually most of your clients are over 60, then in 5 years, they will all be over 65 (MS01).

This is addressed in current work (Carter et al., 2018; Young Dementia UK, 2017) and is indicative of developments that envisage a dementia care pathway that would not regard age >65 years to be a criterion for service discontinuation, but endeavour to provide support and continuity from diagnosis to end-of-life care.

Interviews with community-based third-sector organisations acknowledged the topics addressed in the literature and by PPI discussants and commented on barriers to service delivery. As one organisation’s representative stated, *“…I think we’ve got 42 people with younger onset now, and we’ve got a waiting list that we’ve had for about six months. We’re oversubscribed”* (TSO05). In contrast, one organisation’s service model was based on offering personalised care on a long-term basis, “…*sometimes up to 10 or 12 years*” (TSO10). They adjusted support periodically as determined by the illness trajectory. A key component of this model was that staff were trained and employed on a permanent basis rather than on short-term contracts. Some third-sector organisations offered activities such as walking, gardening, cooking, supper clubs, coffee chats and opportunities to get together socially to share ideas around advocacy (TSO02–TSO05, TSO07 and TSO09-TSO10). However, most services depended on project-based commissioning (TSO02-TSO05 and TSO09), which in turn worked against being able to attract and retain a skilled workforce (TSO05) and serving geographically dispersed populations. This had an impact on the stability and sustainability of provision and on the continuity of care.

Interviews with service providers show that some good quality age-appropriate services are being offered, but often only regionally and therefore not accessible more widely for people with YOD. Service providers recognised the importance of continuity, but were under resourced. With the exception of one charity that offers bespoke long-term care (TS010), service providers did not discuss the potential to create opportunities to offer peer-to-peer support, or how to help families affected by YOD to find alternative support solutions.

## Discussion

Discussions with PPI contributors affected by YOD corroborated findings in national and international literature, but went beyond the consensus that details the challenges of post-diagnostic services for families affected by YOD. This study demonstrated the value of holding discussions with people affected by dementia who can comment on the research findings and provide both a commentary and a critique to existing service solutions. Although they did not articulate this as co-production, their suggestions demonstrated how their experience and expertise could inform service planning. PPI contributors recognised that, because of the small numbers of people with YOD, they may not be able to influence services at the systems level, for example, diagnostic processes and services offered by health and social care, but they saw the potential of changing the focus, support and scope of community-based services through working with local charities and existing services.

Complementary to the findings from the scoping review and third-sector organisations, PPI discussants provided a critical account of what is possible. They offered a framework against which service models could be designed and evaluated, for example, the ability of services to create networks of support that can contribute beyond the lifetime of an activity. PPI contributors saw social networks as being conducive to forming a post-diagnostic identity, establishing new social connections and developing and maintaining a sense of belonging, an approach that has been reported as effective for people affected by YOD (Davies-Quarrell et al., 2010; Gitlin et al., 2015; Roach & Drummond, 2014). These were components of care that were perceived as most desirable and effective, not only for the benefit of the person diagnosed with YOD and their family caregiver, but also as an opportunity for their children to meet peers with a parent in a similar situation. PPI contribution provided invaluable insights on the relative importance of various solutions.

As assessment and diagnostic procedures improve and more people with YOD become known to primary and secondary care services (Young Dementia UK, 2017), increasing numbers will, inevitably, be referred back to the community. Involving families affected by younger onset dementia in networks of support and co-designing locally based community services might address some of the seemingly intractable challenges of sustainability, continuity and cost effectiveness of person-centred and post-diagnostic service provision for this underserved population. Long-term support becomes increasingly important when families’ support needs change as the illness progresses. Continuity of care was rare, yet this was what PPI discussants who were diagnosed some years ago felt was lacking the most.

As highlighted by PPI contributors affected by YOD, a service model that develops cross-cutting links between service providers might foster group-based peer support, enable families in similar situations to support each other and support the organic development of community-based networks. However, this is not to argue that there is no need for additional, ongoing, funded post-diagnostic support for this group. The literature and PPI discussion groups were unequivocal about this.

What this study demonstrates is the importance and potential of involving people with YOD and their families at the centre of the commissioning, design and delivery of dementia care.

### Strengths and limitations of this study

A strength of this study is that it included PPI contributors affected by younger onset dementia to comment on the scoping review and to help shape the questionnaires for third-sector organisations locally. It is worth noting the subtle distinction between PPI contributors and participants and the purpose to which information is used. The blurring of boundaries between the ‘dual role’ of participant and PPI contributor has been reported before (Wilson et al., 2015), is recognised in the current body of PPI literature (Keenan et al., 2017; Pandya-Wood, Barron, & Elliott, 2017; Swaffer, 2016) and raises specific ethical issues within dementia research, as PPI does not require ethical consent (INVOLVE, 2016). There is a need to clarify how this information is systematically collected and organised to ensure that people with YOD have the opportunity to debate and challenge service planning and delivery. A limitation of this study is that PPI contributors were drawn from a pool of people who attend Alzheimer Society’s support groups. Individuals attending such groups are often highly engaged and connected, but the voices of people who do not attend such groups might be heard less. Future studies may need to consider a wider recruitment strategy. However, according to grey literature and the evidence in peer-reviewed literature (Mayrhofer et al., 2017), the issues raised by the four PPI groups in this study were experienced across a wide range of samples of younger people living with dementia and their family caregivers.

## Conclusion

This paper makes an important contribution to the international discourse on dementia care in that it departs from ‘needs based’ reporting and adds to the emerging literature around involving families affected by YOD in the development and co-design of community-based services (Gove et al., 2017; Swarbrick et al., 2016; Tan & Szebeko, 2009). This paper has demonstrated how people with YOD can provide a critical commentary on research findings, current approaches to service provision, and on priorities for future work that complements the evidence and commissioning agenda.

PPI discussants identified the importance of local groups and peer support networks based on reciprocity, but needed help in setting them up, particularly in the initial period. The Dementia Engagement and Empowerment Project (http://dementiavoices.org.uk) is one model that has been instrumental in setting up regional groups, but local charities are needed to help introduce families to each other locally. Research is needed to understand the impact of this kind of approach and how involving people with YOD in priority setting and service design over time affects people’s ability to maintain inclusion.
